# Impact of preoperative thrombocytosis on prognosis after surgical treatment in pathological T1 and T2 renal cell carcinoma: results of a multi-institutional comprehensive study

**DOI:** 10.18632/oncotarget.16136

**Published:** 2017-03-11

**Authors:** Yun-Sok Ha, Jae-Wook Chung, So Young Chun, Seock Hwan Choi, Jun Nyung Lee, Bum Soo Kim, Hyun Tae Kim, Tae-Hwan Kim, Seok-Soo Byun, Eu Chang Hwang, Seok Ho Kang, Sung-Hoo Hong, Jinsoo Chung, Cheol Kwak, Yong-June Kim, Tae Gyun Kwon, KORCC study group

**Affiliations:** ^1^ Department of Urology, School of Medicine, Kyungpook National University, Daegu, Korea; ^2^ Department of Urology, Seoul National University College of Medicine, Seoul National University Bundang Hospital, Seongnam, Korea; ^3^ Department of Urology, Chonnam National University Hwasun Hospital, Jeonnam, Korea; ^4^ Department of Urology, Korea University School of Medicine, Seoul, Korea; ^5^ Department of Urology, College of Medicine, The Catholic University of Korea, Seoul, Korea; ^6^ Department of Urology, National Cancer Center, Goyang, Korea; ^7^ Department of Urology, Seoul National University College of Medicine, Seoul, Korea; ^8^ Department of Urology, Chungbuk National University College of Medicine, Cheongju, Korea

**Keywords:** renal cell carcinoma, prognosis, thrombocytosis

## Abstract

**Background:**

The prognostic significance of preoperative thrombocytosis (TC) in renal cell carcinoma (RCC) is not without some debate. The aim of the present multi-institutional study was to determine the association of preoperative TC with the clinicopathological features and prognosis of localized RCC patients who underwent surgery in a large cohort.

**Methods:**

A study involving 8 institutions, and 4,376 patients with pT1 and pT2 RCC from the Korean renal cell carcinoma (KORCC) database, was conducted. TC was defined as a platelet count ≥400,000/μL. Patients were divided into 2 groups based on the presence of preoperative TC. Clinicopathological variables and survival rates were compared between the 2 groups.

**Results:**

Out of the 4,376 patients in the study, 106 (2.4%) had preoperative TC. Compared to patients without TC, these patients had a lower body mass index. Additionally, these patients had more advanced stage tumors with a higher Fuhrman grade, and higher incidence of symptoms at the time of diagnosis. Kaplan-Meier curves revealed that patients with TC had a significantly lower rate of recurrence-free survival (RFS). Furthermore, a lower rate of overall survival (OS) was exhibited amongst patients with TC. Multivariate analysis revealed that TC was an independent prognostic factor in terms of the RFS and OS.

**Conclusions:**

TC appeared to be an important prognostic determinant in localized RCC. Furthermore, preoperative platelet count may be clinically useful for risk stratification of patients with surgically treated localized RCC.

## INTRODUCTION

In 2016, the estimated numbers of new cases and deaths from kidney cancer were 62,700 and 14,200, respectively, in the United States.[1] Renal cell carcinoma (RCC) accounts for 85% of malignant kidney tumors and has increased annually in the past 2 decades.[2–5] Numerous factors have been implicated in RCC tumorigenesis: chemical carcinogens, lifestyle-associated chronic diseases, and genetic variation.[6] Similar to other tumor entities, RCC manifests itself with significant clinical heterogeneity. Moreover, it can range from indolent to highly aggressive.[7–10] Stratifying a patient's risk of death and disease recurrence has implications for patient counseling and surveillance protocols. Several prognostic parameters have been evaluated and remain important in localized RCC: grade, histologic subtype, and stage.[11–13]

Thrombocytosis (TC), particularly of the reactive or secondary type, has been regarded as a poor prognostic factor in many malignant diseases. These are diseases included: gastric, gynecologic, and lung cancer.[14–17] The possible mechanisms include an overproduction of cytokines and further growth factors stimulating megakaryocytes, in addition to their precursors. Furthermore, there is evidence that platelets might protect circulating tumor cells from detection by the immune system.[18] TC has also been investigated as a valuable prognostic factor for survival and recurrence in RCC patients.[19–22] However, the results of current studies dealing with this subject are conflicting. Thus, the effect of TC on the prognosis of patients with localized RCC (pT1 and pT2) remains unclear.

To address this issue, a large multicenter group of patients with localized RCC, who underwent a radical nephrectomy or a partial nephrectomy, was analyzed for the first time. This is the largest studied group published to date. This study revealed that TC correlated strongly with negative outcomes in patients with localized RCC.

## RESULTS

The mean age of the patients studied was 54.9±13.0 years. There were 106 patients (2.4%) with preoperative TC. Patients with TC had a lower body mass index (BMI) (*P* < 0.001), more symptomatic (*P* = 0.003), a higher T stage (*P* < 0.001), and a higher Fuhrman grade (*P*< 0.001) compared to those without TC (Table 1). Compared to patients without TC, those who had TC were more frequently localized to the renal hilum (*P* = 0.004). Moreover, Preoperative TC showed significant correlation with sarcomatoid differentiation (*P*= 0.001) (Table 1). Patients with TC were treated with a radical nephrectomy more frequently than those without TC (68.9 *vs*. 51.0 %; *P <* 0.001). There were no significant differences between the two groups concerning demographic findings: age, gender, hypertension, diabetes mellitus, and performance status. Regarding histologic type, the TC group had a higher percentage of non-clear cell histology; however, there was no statistical significance.

**Table 1:** Clinicopathological characteristics of the patients divided according to their platelet counts

	Patients without thrombocytosis (N= 4270)	Patients with thrombocytosis (N= 106)	*P*
Mean age ±SD	54.9 ± 12.9	52.8 ± 16.4	0.187
Gender (%)			0.243
Male	2943 (68.9)	67 (63.2)	
Female	1327 (31.1)	39 (36.8)	
BMI, kg/m^2^	24.5 ± 3.4	22.8 ± 3.4	<0.001
Symptom presentation (%)			<0.001
Incidental	3530 (82.7)	70 (66.0)	
Symptomatic	740 (17.3)	36 (34.0)	
Hypertension (%)	1635 (38.3)	34 (32.1)	0.224
DM (%)	580 (13.6)	17 (16.0)	0.437
Type of surgery (%)			<0.001
Radical nephrectomy	2176 (51.0)	73 (68.9)	
Partial nephrectomy	2094 (49.0)	33 (31.1)	
Tumor location			0.004
Exophytic	2132 (49.9)	62 (58.5)	
Mesophytic	728 (17.0)	15 (14.2)	
Endophytic	1008 (23.6)	12 (11.3)	
Hilar	402 (9.4)	17 (16.0)	
Sarcoimatoid differentiation			0.001
No	4214 (98.7)	99 (93.4)	
Yes	56 (1.3)	7 (6.6)	
Histology (%)			0.110
Clear cell	3412 (79.9)	78 (73.6)	
Non-clear cell	858 (20.1)	28 (26.4)	
Pathological T stage (%)			<0.001
T1a	2844 (66.6)	36 (34.0)	
T1b	1038 (24.3)	32 (30.2)	
T2a	280 (6.6)	26 (24.5)	
T2b	108 (2.5)	12 (11.3)	
Fuhrman nuclear grade (%)			<0.001
G1	255 (6.1)	5 (4.7)	
G2	2237 (53.7)	41 (38.7)	
G3	1530 (36.8)	38 (35.8)	
G4	141 (3.4)	22 (20.8)	
Recurrence	282 (6.6)	24 (22.6)	<0.001
Overall death	171 (4.0)	17 (16.0)	<0.001

DM, diabetes mellitus; SD, standard deviation

**Figure 1:**
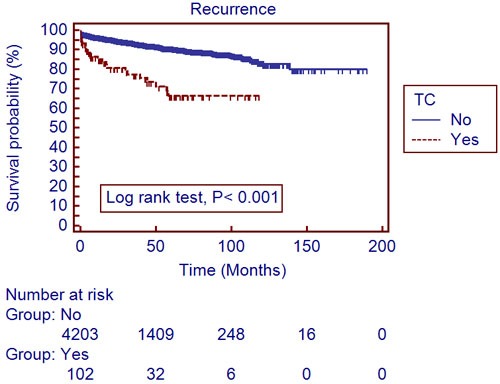
Recurrence-free survival in 4,376 localized renal cell carcinoma patients with and without thrombocytosis (Kaplan-Meier plot); TC, thrombocytosis

**Figure 2:**
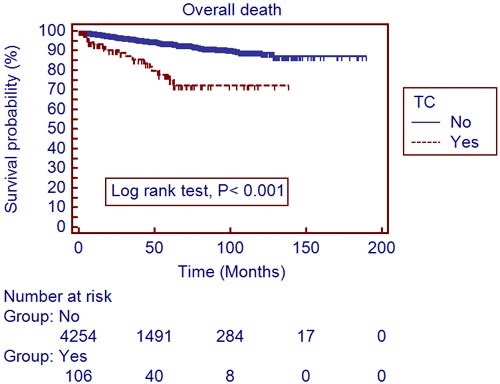
Overall survival in 4,376 localized renal cell carcinoma patients with and without thrombocytosis (Kaplan-Meier plot); TC, thrombocytosis

**Figure 3:**
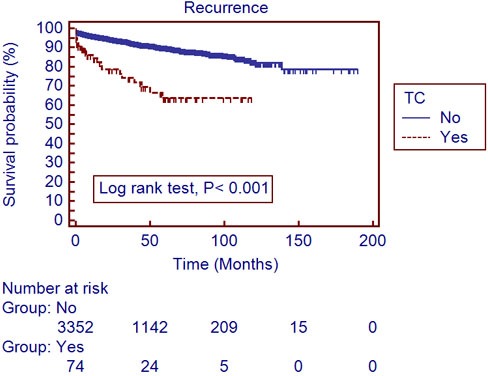
Recurrence-free survival in 3,490 clear cell type renal cell carcinoma patients with and without thrombocytosis (Kaplan-Meier plot); TC, thrombocytosis

**Figure 4:**
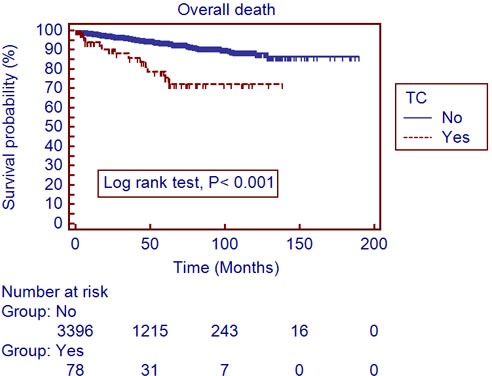
Overall survival in 3,490 clear cell type renal cell carcinoma patients with and without thrombocytosis (Kaplan-Meier plot); TC, thrombocytosis

The mean follow-up period was 40.4±34.6 months. A total of 306 patients (7.0%) developed recurrences and 188 (4.3%) died during the follow-up period. The recurrent free survival (RFS) and overall survival (OS) were significantly lower in patients with TC, compared to those without (Table 1). Utilizing a univariate log-rank test in the Kaplan-Meier analysis, showed RFS (Figure 1; *P* < 0.001) and OS (Figure 2; *P* < 0.001) as significantly associating with preoperative TC. After subgroup analyses of clear cell type RCC, RFS (Figure 3; *P* < 0.001) and OS (Figure 4; *P* < 0.001) were significantly different between the patients with TC and those without TC. Accounting for age, gender, body mass index, pathological T stage, Fuhrman grade, and symptom presentation at diagnosis and operative methods, the Cox proportional hazards model for cancer-specific survival rates showed preoperative TC as an independent prognostic factor for RFS (Table 2; Hazard ratio [HR], 1.927; 95% confidence interval [CI], 1.251-2.971; *P* = 0.003) and OS (Table 3; HR, 2.533; 95% CI, 1.502-4.272; *P* <0.001).

**Table 2:** Multivariate Cox regression analyses of factors that influence recurrence-free survival in localized renal cell carcinoma

	Hazard ratio	95% confidence interval		*P*
		Lower	upper	
Age	1.015	1.005	1.024	0.002
Gender (Male *vs*. Female)	0.701	0.538	0.913	0.008
Body mass index	0.968	0.934	1.003	0.073
Symptom (No *vs*. Yes)	1.504	1.166	1.941	0.002
Thrombocytosis (No *vs*. Yes)	1.927	1.251	2.971	0.003
T stage (T1 *vs*. T2)	1.945	1.701	2.225	<0.001
Fuhrman grade (G1-2 *vs*. G3-4)	2.048	1.718	2.441	<0.001
Operative methods (Radical *vs*. Partial)	0.799	0.578	1.104	0.174

**Table 3:** Multivariate Cox regression analyses of factors that influence overall survival in localized renal cell carcinoma

	Hazard ratio	95% confidence interval		*P*
		Lower	upper	
Age	1.041	1.028	1.055	<0.001
Gender (Male *vs*. Female)	0.539	0.377	0.772	0.001
Body mass index	0.908	0.866	0.952	<0.001
Symptom (No *vs*. Yes)	1.394	1.007	1.929	0.045
Thrombocytosis (No *vs*. Yes)	2.533	1.502	4.272	<0.001
T stage (T1 *vs*. T2)	1.719	1.450	2.038	<0.001
Fuhrman grade (G1-2 *vs*. G3-4)	1.774	1.420	2.217	<0.001
Operative methods (Radical *vs*. Partial)	0.464	0.288	1.104	0.002

## DISCUSSION

The present study examined data from a large, multi-institutional cohort of patients with localized RCC and found that patients with TC had significantly lower RFS and OS than patients without TC, even after adjusting for other known predictors of RCC prognosis. Several studies have evaluated the association between TC and RCC prognosis. However, the results were not consistent and the prognostic significance of TC in RCC is still a subject of debate. Some researchers reported a positive correlation between RCC prognosis and TC.[19, 20, 22–27] Nevertheless, other studies reported that TC was not an independent predictor of prognosis in multivariate models.[21, 28, 29] Due to the relatively small number of cases in previous studies, it would have been hasty to conclude that there was an association between TC and RCC prognosis. Furthermore, study populations in previous reports were not homogenous, because they included all stages. Among them, Choi et al. conducted a retrospective analysis of 187 patients who underwent a radical nephrectomy for non-metastatic RCC.[5] They included about 90% T1 and T2 patients. The study of Inoue et al. showed that TC was an independent predictor of CSS only in stage 1 and 2 RCC.[27] On the contrary, the strength of our current series was that we had the largest study cohort ever, and included only pT1 and pT2 RCC. This was to ensure a homogeneous study population.

In the present study, patients who had both RCC and TC had a lower BMI than those without TC. They also had a higher incidence of symptoms at diagnosis, a higher T stage, and a higher Fuhrman grade, than those without TC. TNM classification, Fuhrman nuclear grade, and symptoms at diagnosis were previously evaluated prognostic markers.[12] In addition, the nutritional and immunologic status of cancer patients could affect the perioperative outcome and survival associated with malignant tumors. Haferkamp et al. reported that being underweight (BMI <18.5 kg/m^2^) is an unfavorable factor for patient outcomes after nephrectomy for RCC.[30] Consistent with previous results, BMI was also an independent prognosticator in localized RCC. Additionally, low BMI was significantly correlated with TC. Taken together and in terms of baseline characteristics, these differences between patients with and without TC may explain why those with TC in our cohort had a more aggressive disease.

TC is known to correlate with poor clinical outcomes in cancer and is known to be caused by tumor cells. Additionally platelets are known to participate in metastasis. More than 45 years ago, it was established that thrombocytopenic mice were protected against metastasis.[31] Since then, extensive data has supported the relevance of platelets in the progression of cancer.[32] Janowska-Wieczorek et al. showed that platelet-derived microparticles stimulate mitogen-activated protein kinases in lung carcinoma cell lines and increase cell proliferation.[33] Platelets induce matrix metalloproteinase (MMP)-9 expression and activation in the cell lines of colon and breast cancer. This leads to an increased remodeling of the extracellular matrix, release of growth factors from the extracellular matrix, and relief of cell-cell contacts. All of these factors decrease apoptotic signals.[34] In a cohort of patients with ovarian cancer, elevated platelet counts correlated with decreased overall survival and resistance to chemotherapy.[35, 36]

Platelets also have the ability to deliver multiple proangiogenic factors to the tumor. Moreover, platelets also have the ability to stimulate expression of proangiogenic factors by the tumor cell.[33] Platelets have long been identified as a major source of vascular endothelial growth factor (VEGF),[37, 38] a platelet-derived growth factor (PDGF), and a basic fibroblast growth factor (bFGF), each of which promotes tumor growth.[39, 40] Activation of platelets and the release of platelet microparticles, leads to the release of a variety of proangiogenic factors. These factors include, VEGF, PDGF, FGF, and MMPs. Increased levels of platelet microparticles are found in the plasma of patients with both solid tumors and hematologic malignancies.[41] In those with gastric cancer, the highest circulating levels of platelet microparticles were found in individuals with stage IV of the disease. These levels significantly correlated with metastatic disease.[42] Platelets also play a role in osteolytic, bone metastatic, breast cancer. Boucharaba et al. reported that platelet-derived lysophosphatidic acid (LPA) can support and stimulate metastatic breast cancer cells.[43] High platelet count is associated with increased mortality in a variety of cancers: malignant mesothelioma, gynecological malignancies, lung, renal, gastric, colorectal, and breast.[15, 17, 35, 44–49]

The limitations of the current study include a relatively short follow-up period and the retrospective nature of the data collection. A short follow-up might affect subsequent conclusions. Moreover, no information was available concerning inflammatory laboratory parameters such as, the neutrophil-to-lymphocyte ratio and the platelet-to-lymphocyte ratio, etc. To overcome these concerns, investigators from the centers involved in this research will follow up the study population continuously, and on a longitudinal basis. The other concern was that the frequency of TC was too small only in 2.4%. It means that the assessment of platelets counts may be useful in small portion of cohort. Lastly, our multi-institutional database did not contain platelet count after surgery. In near future, we will analyze the platelet count differences between before and after surgery.

In conclusions, there was a significant correlation between TC and tumor-related factors. These factors included pathological T stage, Fuhrman grade, and symptoms at the time of diagnosis. TC was a significant predictor of RFS and OS in patients with pT1-2 RCC. Therefore, TC might be a useful tool to predict survival after nephrectomy in localized RCC patients. Moreover, patients with TC may be stratified to undergo adjuvant targeted therapy and more frequent follow-up strategies should be need in these patient groups.

## MATERIALS AND METHODS

Between 1999 and 2015 an IRB approved study, involving 8 institutions and 7,136 patients with pT1 and pT2 RCC from the Korean renal cell carcinoma (KORCC) database, was conducted.[50–52] Patients with stage pT3 or greater cancer, lymph-node or distant metastases, and low platelet counts (less than 130,000/μL) were excluded from the study. We also excluded patients who took medication that might cause thrombocytosis and those who had hematologic disorders. As a result, 4,376 patients with localized RCC (pT1 and pT2) were included in the study. All patients were examined with routine hematologic laboratory tests. Additionally, radiologic imaging, including chest X-rays and abdominal computed tomography, was utilized. TC was defined as a platelet count ≥400,000/μL. Patients were divided into 2 groups based on the presence of preoperative TC.[5, 20] This cut-off value was based on the thresholds given by the participating institute's laboratories. Moreover, this value has been used in most studies concerning this topic. The staging manual, from The American Joint Committee on Cancer, was used for TNM staging. Nuclear differentiation was evaluated using the Fuhrman grading system.[53, 54] All patients were evaluated postoperatively: every 3 months for the first 2 years, every 6 months for the next 2 years, and yearly thereafter. These evaluations included physical examinations, and radiologic investigations. Recurrence was defined as local relapse, lymph-node metastasis, or distant metastasis. Recurrence was identified by an abdominal and thoracic investigation (bone scan and computed tomography).

Pertinent characteristics of patients, with and without TC, were compared. The Shapiro-Wilk normality test was used to investigate the normal distribution of continuous variables. Continuous, evenly distributed variables, were reported as average with standard deviation. Student's t-test was applied for normally distributed variables. Comparison between categorical variables was performed using Fisher's exact test and the chi-square test. The survival distributions, including patient survival and tumor recurrence, were estimated by the Kaplan-Meier method. Comparison of survival distributions was done by a log-rank test. Furthermore, the data was censored at the time of the last visit. The effect of TC on RFS and OS was examined using a Cox proportional hazard regression model. The HR and 95-% CI were computed. Data was analyzed using SPSS 18.0 (SPSS Inc., Chicago, IL, USA). *P* values were two-sided with the statistical significance level set at *P* ≤ 0.05.

## References

[1] Siegel RL, Miller KD, Jemal A (2016). Cancer statistics, 2016. CA Cancer J Clin.

[2] Kang HW, Seo SP, Kim WT, Yun SJ, Lee SC, Kim WJ, Hwang EC, Kang SH, Hong SH, Chung J, Kwon TG, Kim HH, Kwak C (2016). Impact of Young Age at Diagnosis on Survival in Patients with Surgically Treated Renal Cell Carcinoma: a Multicenter Study. J Korean Med Sci.

[3] Lindblad P (2004). Epidemiology of renal cell carcinoma. Scand J Surg.

[4] Verhoest G, Veillard D, Guille F, De La Taille A, Salomon L, Abbou CC, Valeri A, Lechevallier E, Descotes JL, Lang H, Jacqmin D, Tostain J, Cindolo L (2007). Relationship between age at diagnosis and clinicopathologic features of renal cell carcinoma. Eur Urol.

[5] Choi JY, Ko YH, Song PH (2016). Clinical significance of preoperative thrombocytosis in patients who underwent radical nephrectomy for nonmetastatic renal cell carcinoma. Investig Clin Urol.

[6] Cho E, Adami HO, Lindblad P (2011). Epidemiology of renal cell cancer. Hematol Oncol Clin North Am.

[7] Kim YW, Kim WT, Yun SJ, Lee SC, Kim WJ, Ha YS, Park YH, Kang SH, Hong SH, Kwon TG, Byun SS, Kwak C, Kim YJ (2015). Preoperative Chronic Kidney Disease Status is an Independent Prognostic Factor in Patients with Renal Cell Carcinoma. Ann Surg Oncol.

[8] Ha YS, Lee GT, Modi P, Kwon YS, Ahn H, Kim WJ, Kim IY (2015). Increased Expression of Androgen Receptor mRNA in Human Renal Cell Carcinoma Cells is Associated with Poor Prognosis in Patients with Localized Renal Cell Carcinoma. J Urol.

[9] Ha YS, Lee GT, Kim YH, Kwon SY, Choi SH, Kim TH, Kwon TG, Yun SJ, Kim IY, Kim WJ (2014). Decreased selenium-binding protein 1 mRNA expression is associated with poor prognosis in renal cell carcinoma. World J Surg Oncol.

[10] Ha YS, Chihara Y, Yoon HY, Kim YJ, Kim TH, Woo SH, Yun SJ, Kim IY, Hirao Y, Kim WJ (2013). Downregulation of fumarate hydratase is related to tumorigenesis in sporadic renal cell cancer. Urol Int.

[11] Van Brussel JP, Mickisch GH (1999). Prognostic factors in renal cell and bladder cancer. BJU Int.

[12] Ficarra V, Righetti R, Pilloni S, D'Amico A, Maffei N, Novella G, Zanolla L, Malossini G, Mobilio G (2002). Prognostic factors in patients with renal cell carcinoma: retrospective analysis of 675 cases. Eur Urol.

[13] Cheville JC, Lohse CM, Zincke H, Weaver AL, Blute ML (2003). Comparisons of outcome and prognostic features among histologic subtypes of renal cell carcinoma. Am J Surg Pathol.

[14] Griesshammer M, Bangerter M, Sauer T, Wennauer R, Bergmann L, Heimpel H (1999). Aetiology and clinical significance of thrombocytosis: analysis of 732 patients with an elevated platelet count. J Intern Med.

[15] Pedersen LM, Milman N (1996). Prognostic significance of thrombocytosis in patients with primary lung cancer. Eur Respir J.

[16] Hernandez E, Lavine M, Dunton CJ, Gracely E, Parker J (1992). Poor prognosis associated with thrombocytosis in patients with cervical cancer. Cancer.

[17] Zeimet AG, Marth C, Muller-Holzner E, Daxenbichler G, Dapunt O (1994). Significance of thrombocytosis in patients with epithelial ovarian cancer. Am J Obstet Gynecol.

[18] Nieswandt B, Hafner M, Echtenacher B, Mannel DN (1999). Lysis of tumor cells by natural killer cells in mice is impeded by platelets. Cancer Res.

[19] Wosnitzer M, Polland A, Hai Q, Hruby G, McKiernan J (2011). Role of preoperative platelet level in clinical and pathological outcomes after surgery for renal cortical malignancies. BJU Int.

[20] Brookman-May S, May M, Ficarra V, Kainz MC, Kampel-Kettner K, Kohlschreiber S, Wenzl V, Schneider M, Burger M, Wieland WF, Otto W, Tilki D, Gilfrich C (2013). Does preoperative platelet count and thrombocytosis play a prognostic role in patients undergoing nephrectomy for renal cell carcinoma? Results of a comprehensive retrospective series. World J Urol.

[21] Cho DS, Kim SJ, Lee SH, Ahn HS, Kim YS, Kim SI (2011). Prognostic significance of preoperative C-reactive protein elevation and thrombocytosis in patients with non-metastatic renal cell carcinoma. Korean J Urol.

[22] Gogus C, Baltaci S, Filiz E, Elhan A, Beduk Y (2004). Significance of thrombocytosis for determining prognosis in patients with localized renal cell carcinoma. Urology.

[23] Brookman-Amissah S, Kendel F, Spivak I, Pflanz S, Roigas J, Klotz T, May M (2009). Impact of clinical variables on predicting disease-free survival of patients with surgically resected renal cell carcinoma. BJU Int.

[24] Karakiewicz PI, Trinh QD, Lam JS, Tostain J, Pantuck AJ, Belldegrun AS, Patard JJ (2007). Platelet count and preoperative haemoglobin do not significantly increase the performance of established predictors of renal cell carcinoma-specific mortality. Eur Urol.

[25] Bensalah K, Leray E, Fergelot P, Rioux-Leclercq N, Tostain J, Guille F, Patard JJ (2006). Prognostic value of thrombocytosis in renal cell carcinoma. J Urol.

[26] Erdemir F, Kilciler M, Bedir S, Ozgok Y, Coban H, Erten K (2007). Clinical significance of platelet count in patients with renal cell carcinoma. Urol Int.

[27] Inoue K, Kohashikawa K, Suzuki S, Shimada M, Yoshida H (2004). Prognostic significance of thrombocytosis in renal cell carcinoma patients. Int J Urol.

[28] Ito K, Asano T, Yoshii H, Satoh A, Sumitomo M, Hayakawa M (2006). Impact of thrombocytosis and C-reactive protein elevation on the prognosis for patients with renal cell carcinoma. Int J Urol.

[29] Lee SE, Byun SS, Han JH, Han BK, Hong SK (2006). Prognostic significance of common preoperative laboratory variables in clear cell renal cell carcinoma. BJU Int.

[30] Haferkamp A, Pritsch M, Bedke J, Wagener N, Pfitzenmaier J, Buse S, Hohenfellner M (2008). The influence of body mass index on the long-term survival of patients with renal cell carcinoma after tumour nephrectomy. BJU Int.

[31] Gasic GJ, Gasic TB, Stewart CC (1968). Antimetastatic effects associated with platelet reduction. Proc Natl Acad Sci U S A.

[32] Gay LJ, Felding-Habermann B (2011). Contribution of platelets to tumour metastasis. Nat Rev Cancer.

[33] Janowska-Wieczorek A, Wysoczynski M, Kijowski J, Marquez-Curtis L, Machalinski B, Ratajczak J, Ratajczak MZ (2005). Microvesicles derived from activated platelets induce metastasis and angiogenesis in lung cancer. Int J Cancer.

[34] Labelle M, Begum S, Hynes RO (2011). Direct signaling between platelets and cancer cells induces an epithelial-mesenchymal-like transition and promotes metastasis. Cancer Cell.

[35] Stone RL, Nick AM, McNeish IA, Balkwill F, Han HD, Bottsford-Miller J, Rupairmoole R, Armaiz-Pena GN, Pecot CV, Coward J, Deavers MT, Vasquez HG, Urbauer D (2012). Paraneoplastic thrombocytosis in ovarian cancer. N Engl J Med.

[36] Bottsford-Miller J, Choi HJ, Dalton HJ, Stone RL, Cho MS, Haemmerle M, Nick AM, Pradeep S, Zand B, Previs RA, Pecot CV, Crane EK, Hu W (2015). Differential platelet levels affect response to taxane-based therapy in ovarian cancer. Clin Cancer Res.

[37] Verheul HM, Hoekman K, Luykx-de Bakker S, Eekman CA, Folman CC, Broxterman HJ, Pinedo HM (1997). Platelet: transporter of vascular endothelial growth factor. Clin Cancer Res.

[38] Pinedo HM, Verheul HM, D'Amato RJ, Folkman J (1998). Involvement of platelets in tumour angiogenesis?. Lancet.

[39] Cross MJ, Claesson-Welsh L (2001). FGF and VEGF function in angiogenesis: signalling pathways, biological responses and therapeutic inhibition. Trends Pharmacol Sci.

[40] Ferrara N, Gerber HP, LeCouter J (2003). The biology of VEGF and its receptors. Nat Med.

[41] Mezouar S, Mege D, Darbousset R, Farge D, Debourdeau P, Dignat-George F, Panicot-Dubois L, Dubois C (2014). Involvement of platelet-derived microparticles in tumor progression and thrombosis. Semin Oncol.

[42] Kim HK, Song KS, Park YS, Kang YH, Lee YJ, Lee KR, Kim HK, Ryu KW, Bae JM, Kim S (2003). Elevated levels of circulating platelet microparticles, VEGF, IL-6 and RANTES in patients with gastric cancer: possible role of a metastasis predictor. Eur J Cancer.

[43] Boucharaba A, Serre CM, Gres S, Saulnier-Blache JS, Bordet JC, Guglielmi J, Clezardin P, Peyruchaud O (2004). Platelet-derived lysophosphatidic acid supports the progression of osteolytic bone metastases in breast cancer. J Clin Invest.

[44] Costantini V, Zacharski LR, Moritz TE, Edwards RL (1990). The platelet count in carcinoma of the lung and colon. Thromb Haemost.

[45] Ikeda M, Furukawa H, Imamura H, Shimizu J, Ishida H, Masutani S, Tatsuta M, Satomi T (2002). Poor prognosis associated with thrombocytosis in patients with gastric cancer. Ann Surg Oncol.

[46] Kerpsack JT, Finan MA (2000). Thrombocytosis as a predictor of malignancy in women with a pelvic mass. J Reprod Med.

[47] Menczer J, Schejter E, Geva D, Ginath S, Zakut H (1998). Ovarian carcinoma associated thrombocytosis. Correlation with prognostic factors and with survival. Eur J Gynaecol Oncol.

[48] O'Keefe SC, Marshall FF, Issa MM, Harmon MP, Petros JA (2002). Thrombocytosis is associated with a significant increase in the cancer specific death rate after radical nephrectomy. J Urol.

[49] Taucher S, Salat A, Gnant M, Kwasny W, Mlineritsch B, Menzel RC, Schmid M, Smola MG, Stierer M, Tausch C, Galid A, Steger G, Jakesz R (2003). Impact of pretreatment thrombocytosis on survival in primary breast cancer. Thromb Haemost.

[50] Byun SS, Hong SK, Lee S, Kook HR, Lee E, Kim HH, Kwak C, Ku JH, Jeong CW, Lee JY, Hong SH, Kim YJ, Hwang EC (2016). The establishment of KORCC (KOrean Renal Cell Carcinoma) database. Investig Clin Urol.

[51] Ha YS, Kim WT, Yun SJ, Lee SC, Kim WJ, Park YH, Kang SH, Hong SH, Byun SS, Kim YJ (2013). Multi-institutional analysis of localized renal cell carcinoma that demonstrates the impact of diabetic status on prognosis after nephrectomy. Ann Surg Oncol.

[52] Ha YS, Park YH, Kang SH, Hong SH, Hwang TK, Byun SS, Kim YJ (2013). Predictive factors for late recurrence in patients with stage T1 clear cell renal cell carcinoma: a multiinstitutional study. Clin Genitourin Cancer.

[53] Greene FL (2002). The American Joint Committee on Cancer: updating the strategies in cancer staging. Bull Am Coll Surg.

[54] Fuhrman SA, Lasky LC, Limas C (1982). Prognostic significance of morphologic parameters in renal cell carcinoma. Am J Surg Pathol.

